# Conserving Tropical Tree Diversity and Forest Structure: The Value of Small Rainforest Patches in Moderately-Managed Landscapes

**DOI:** 10.1371/journal.pone.0098931

**Published:** 2014-06-05

**Authors:** Manuel A. Hernández-Ruedas, Víctor Arroyo-Rodríguez, Jorge A. Meave, Miguel Martínez-Ramos, Guillermo Ibarra-Manríquez, Esteban Martínez, Gilberto Jamangapé, Felipe P. L. Melo, Bráulio A. Santos

**Affiliations:** 1 Centro de Investigaciones en Ecosistemas, Universidad Nacional Autónoma de México, Morelia, Michoacán, Mexico; 2 Departamento de Ecología y Recursos Naturales, Facultad de Ciencias, Universidad Nacional Autónoma de México, Mexico City, Mexico; 3 Departamento de Botánica, Instituto de Biología, Universidad Nacional Autónoma de México, Mexico City, Mexico; 4 Ejido Loma Bonita, Ocosingo, Chiapas, Mexico; 5 Departamento de Botânica, Universidade Federal de Pernambuco, Recife, Pernambuco, Brazil; 6 Departamento de Sistemática e Ecologia, Universidade Federal da Paraíba, João Pessoa, Paraíba, Brazil; Landcare Research, New Zealand

## Abstract

Rainforests are undergoing severe deforestation and fragmentation worldwide. A huge amount of small forest patches are being created, but their value in conserving biodiversity and forest structure is still controversial. Here, we demonstrate that in a species-rich and moderately-managed Mexican tropical landscape small rainforest patches (<100 ha) can be highly valuable for the conservation of tree diversity and forest structure. These patches showed diverse communities of native plants, including endangered species, and a new record for the country. Although the number of logged trees increased in smaller patches, patch size was a poor indicator of basal area, stem density, number of species, genera and families, and community evenness. Cumulative species-area curves indicated that all patches had a similar contribution to the regional species diversity. This idea also was supported by the fact that patches strongly differed in floristic composition (high β-diversity), independently of patch size. Thus, in agreement with the land-sharing approach, our findings support that small forest patches in moderately-managed landscapes should be included in conservation initiatives to maintain landscape heterogeneity, species diversity, and ecosystem services.

## Introduction

Approximately 83% of earth’s land surface has been altered by human action [1], leading to the rapid destruction and fragmentation of terrestrial ecosystems. In the tropics, habitat loss and degradation are main drivers of biodiversity loss [2], but the effectiveness of forest patches to retain large subsets of species diversity and forest structure is still controversial [3], [4], particularly when considering tropical plants [5], [6]. For example, whereas some studies argue that highly fragmented tropical landscapes can maintain high levels of the original diversity [7], [8], others demonstrate that species diversity in forest patches declines rapidly after forest fragmentation [9], [10].

In particular, increasing tree mortality rates have been reported near forest edges [9], particularly among emergent species [11], [12]. This process may lead to declines in tree species richness in smaller forest patches [10], [13], particularly near forest edges [11], [12]. Logging and tree mortality can also alter forest structure, as they usually provoke the collapse of tree biomass [9]. However, under certain circumstances (e.g., in recently fragmented landscapes with lower deforestation levels), small and large forest patches can maintain similar tree species diversities [7], [8]. Thus, to correctly assess the conservation value of small forest patches it is necessary to conduct more studies, encompassing landscapes and regions with contrasting management histories and intensities of land use. Such studies are particularly valuable if performed within tropical biodiversity hotspots [6], [7], [14].

The Mesoamerican region is among the most important tropical biodiversity hotspots because of their high number of species and high rates of deforestation [15]. The Lacandon rainforest contributes greatly to the floristic diversity of Mesoamerica, especially the Mexican portion of this forest, where ca. 3000 vascular plant species have been recorded [16]. Unfortunately, the rapid loss and fragmentation of this forest are seriously threatening this extraordinary biodiversity. Several studies have described the composition and forest structure of the Mexican Lacandon rainforest [e.g., 16–18]; however, most of them have been carried out in regenerating forest patches or within the Chajul Biological Station; an area of well-preserved continuous forest. Here we assess, for the first time, the value of small old-growth forest patches (<100 ha) for the maintenance of tree diversity and forest structure in the region. Specifically, we evaluate whether tree species diversity and forest structure are related to patch size, and assess the relative contribution of small forest patches to regional diversity.

**Figure 1:**
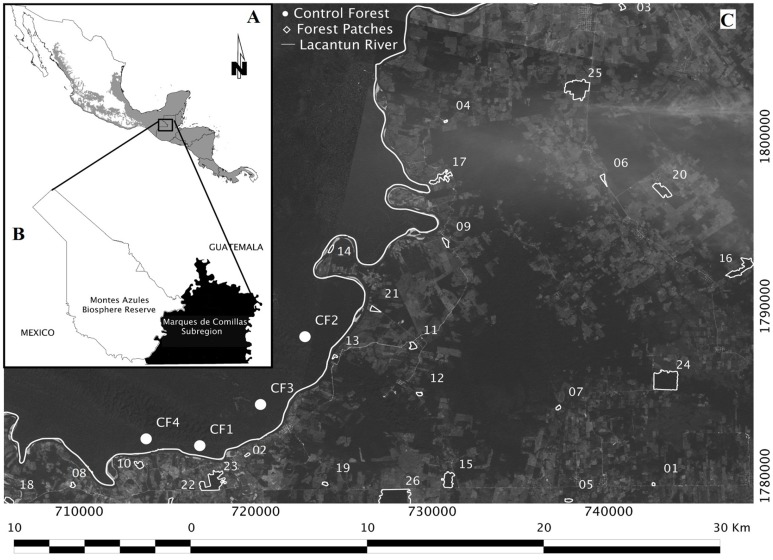
Location of the Mesoamerican biodiversity hotspot (a), and the study area in the Lacandon rainforest, Mexico (b). The 26 forest patches located in the Marqués de Comillas region and the four reference areas studied within the continuous forest of the Montes Azules Biosphere Reserve are also indicated (c).

Evidence indicates that the value of small patches for biodiversity conservation in human-modified landscapes is higher in recently fragmented landscapes [6], with higher remaining forest cover [7], [19], [20], and embedded in a heterogeneous landscape matrix [21], [22]. Biodiversity maintenance is also dependent on the ‘health’ of food webs [4]. Deforestation in our study area is relatively recent (<40 years ago), the remaining forest cover is considerably high (ca. 40%; Figure 1), the matrix surrounding forest patches is very heterogeneous (see Methods), and the region still maintains their original fauna [23]. Within this landscape context, we hypothesized that small rainforest patches would be highly valuable for the conservation of tree diversity and forest structure. This hypothesis implies weak correlations between patch size and vegetation attributes, and a high contribution of small forest patches to regional diversity.

## Materials and Methods

### Ethics Statement

All necessary permits were obtained for this study. It adhered to the laws of the Mexican Government (SEMARNAT, Secretaría de Medio Ambiente y Recursos Naturales) to sample and collect animals and plants in Lacandona (permit no. SGPA/DGVS/09606). Although our institution, Universidad Nacional Autónoma de Mexico (UNAM), does not yet have an Institutional Review Board (IRB) or a similar governing body of ethics, this project was approved by the institutional authorities from UNAM and the Programa de Apoyo a Proyectos de Investigación e Innovación Tecnológica (PAPIIT), DGAPA-UNAM (Projects IA-203111, IB-200812 and RR-280812). The owners of the forest patches gave us the permission to perform the research in the study sites.

### Study Area

The Lacandon rainforest comprises parts of Belize, Guatemala, and Mexico, and is one of the largest areas of tropical rainforest (ca. 800,000 ha) in Mesoamerica. The study was carried out in the Mexican portion of the Lacandon rainforest, Chiapas, Mexico (16°04′–16°21′N, 90°40′–91°06′W; 100–400 m a.s.l.; Figure 1). The climate is warm and humid, with a mean annual temperature of 24.1°C and average annual rainfall of 2875 mm.

In 1978, the Montes Azules Biosphere Reserve (MABR) was created, encompassing 331,200 ha of continuous forest. Outside this reserve, the Marqués de Comillas region (MCR) encompasses 203,999 ha of disturbed forests and human settlements. The dominant vegetation types in both regions are semi-deciduous tropical rainforest and lowland tropical rainforest [16], [24]. During the 1970s, the MCS suffered a colonization process by a multicultural population [25], and it has been largely deforested since, especially between 1984 and 1986 [25], owing to national policies that promoted agroforestry, agriculture, and cattle ranching. The main cause of the massive deforestation in the MCS was the so-called ‘Plan Piloto Forestal de Marqués de Comillas’, resulting in tree down-cutting in 30% of the area during 1998 and 1999 [24]. Nowadays, approximately 40% of old-growth forest cover remains as remnant patches of variable sizes embedded in a heterogeneous matrix of secondary forests, human settlements, cattle pastures, and crop fields.

The study was conducted in two adjacent areas separated by the Lacantún River: the continuous forest of MABR and the fragmented forest of MCR (Figure 1). In the MCR, we selected 26 isolated old-growth forest patches (ranging from 2 to 92 ha). The forest patches were selected according to the following criteria: (i) they represented discrete forest masses separated by cattle pastures and crop fields, (ii) they were separated from each other as much as possible to increase independence among them, and (iii) they were all located between 100 and 250 m a.s.l. to avoid the effects of altitudinal (and soil) gradients. The size of all forest patches was calculated using recent SPOT-5 satellite images (March 2011) and Quantum GIS 1.7.4. (Quantum GIS Development Team, 2012). In addition, as reference sites (100% of forest cover), we selected four buffer areas of 100 ha within the continuous forest of MABR, which were spaced 4 km apart (on average) and at least 1 km from the nearest edge of the Lacantún River (Figure 1).

### Vegetation Sampling

We used Gentry’s [26] protocol to sample vegetation in each site, but recording only tree species (including palms) with a diameter at breast height (dbh) ≥2.5 cm in ten 50×2-m transects randomly located at each site (0.1 ha per site). Unidentified specimens were collected for their identification at the National Herbarium of Mexico (MEXU, Mexico City). Plant names followed the Missouri Botanical Garden electronic database (Tropicos) available at http://www.tropicos.org. To assess differences among sites in tree mortality and logging, we also recorded the number of dead and logged trees within transects.

### Data Analyses

To avoid pseudoreplication problems, we summed up the data of the 10 transects from each site and considered the sites as independent samples for the following analyses. In particular, we recorded the number of families, genera, species, stems, and dead and logged trees within each site. We also estimated the total basal area by summing the basal area of all stems (assuming the stem cross-section area as a circle). To assess the inventory completeness within each site, we employed the coverage estimator suggested by Chao and Jost [27], which is a less biased estimator of sample completeness:
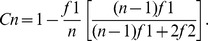
where *f_1_* and *f_2_* are the number of singletons and doubletons in the sample, respectively, and *n* is the number of individuals. Because sample coverage varied among sites (Table 1), our estimates of the number of species, genera and families could be biased by differences in sample completeness [27]. Thus, following Chao & Jost [27] and Chao et al. [28], we estimated the number of species, genera and families in each site using coverage-based rarefaction (interpolation) with the iNEXT software [29]. In particular, we considered the minimum completeness for all sites (0.82 in species, 0.86 in genera and 0.93 in families) to have reliable and comparable estimates of the number of species, genera and families based on samples of equal completeness (equal coverage) [27], [28].

**Table 1:** Number of plant species, genera and families in 26 forest patches (FP) and 4 reference sites within a continuous forest (CF) sampled in the fragmented Lacandon rainforest, Chiapas, Mexico.

Sites	Size (ha)	Species			Genera			Families		
		Obs	*Ĉ_n_*	Exp	Obs	*Ĉ_n_*	Exp	Obs	*Ĉ_n_*	Exp
FP1	2.4	65	0.92	50	56	0.94	45	34	0.97	29
FP2	2.8	38	0.91	27	33	0.93	24	18	0.96	14
FP3	4.8	36	0.94	22	32	0.95	23	26	0.96	22
FP4	5.7	58	0.87	48	51	0.89	45	26	0.97	22
FP5	6	67	0.88	54	55	0.91	46	33	0.97	29
FP6	12.7	58	0.89	49	49	0.93	43	27	0.98	25
FP7	12.7	72	0.86	68	59	0.90	56	29	0.96	26
FP8	14.5	58	0.92	45	51	0.93	43	27	0.99	23
FP9	18	43	0.85	39	36	0.89	34	22	0.96	20
FP10	20.3	66	0.89	52	50	0.93	39	31	0.97	27
FP11	20.6	68	0.89	58	57	0.91	50	35	0.96	32
FP12	22.4	62	0.85	55	54	0.89	49	32	0.93	32
FP13	28.1	68	0.84	62	59	0.86	59	35	0.94	32
FP14	33	31	0.91	14	26	0.93	15	19	0.97	16
FP15	33.2	76	0.88	64	61	0.92	53	34	0.97	30
FP16	33.2	55	0.90	44	46	0.93	39	29	0.97	26
FP17	33.4	59	0.83	57	47	0.88	45	26	0.95	25
FP18	37.8	59	0.87	55	51	0.90	49	28	0.99	27
FP19	37.8	75	0.82	75	64	0.86	64	35	0.97	31
FP20	42.3	69	0.86	62	57	0.90	51	29	0.97	26
FP21	51.3	47	0.92	39	43	0.93	38	22	0.99	19
FP22	62.9	64	0.88	51	49	0.92	39	33	0.96	30
FP23	65.5	63	0.89	50	53	0.92	44	25	0.97	19
FP24	72.1	60	0.89	43	48	0.93	36	29	0.97	24
FP25	75.9	58	0.90	41	51	0.92	40	28	0.97	24
FP26	91.9	52	0.92	30	45	0.94	31	24	0.99	20
CF1	100	74	0.82	74	61	0.90	58	30	0.98	27
CF2	100	51	0.89	35	38	0.93	27	25	0.97	20
CF3	100	70	0.85	64	57	0.86	57	33	0.95	30
CF4	100	55	0.88	47	46	0.92	41	24	0.98	21

The observed values (Obs) and the expected (Exp) after a coverage-based rarefaction (interpolation) are indicated for each site. The coverage estimator suggested by Chao and Jost [27] is also indicated for each case (*Ĉ_n_*, see Methods).

We also assessed changes in community evenness among sites using the evenness factor (*EF*) proposed by Jost [30]: *EF* = *^2^D/S*, where *^2^D* is the inverse Simpson concentration and *S* is the total number of species in the sample. This index ranges between 1 (all species are equally common) and nearly 1/*S* (the community is dominated by one species [30]). Roughly, *EF* is interpreted as the proportion of dominant species in the community [30].

To test whether tree species diversity and forest structure were related to patch size, we performed simple linear regression models between patch size (log_10_-transformed), taken as the independent variable, and all vegetation attributes (i.e., rarefied number of species, genera, families, density of stems and dead and logged trees, total basal area, and *EF* per site), taken as response variables. Reference sites were included in the models assuming that they covered 100 ha. To evaluate the changes in species composition across sites, we estimated two measures of compositional similarity, the Jaccard index based on the presence and absence of each species, and the abundance-based Bray-Curtis index. Then, we performed a non-metric multidimensional scaling (NMDS) using the Bray-Curtis index to assess the overall differences in species composition among sites. The sites were classified based on different size classes (<10 ha, 10–30 ha, >30–50 ha, >50–100 ha, and continuous forest sites), and a multivariate analysis of variance (MANOVA) was performed to test for significant differences among size categories over the ordination dimensions generated by the NMDS analysis. We also used Mantel tests to assess whether variations in floristic composition between sites (Bray-Curtis index) were related to patch location (i.e., log-transformed inter-site isolation distances) and/or to differences between sites in size. *P*-values were calculated using the distribution of the *R* coefficients obtained from 10,000 permutations.

Finally, to assess the contribution of small forest patches to regional diversity, we used cumulative species-area curves [7], [31]. The sites were ordered based on their area from small to large, and then from large to small [32]. We plotted the cumulative number of species versus the number of sites added to compare the cumulative observed number of species in the small to large and large to small curves. To evaluate the effect of habitat subdivision on tree species diversity, we calculated the Saturation Index (SI) of Quinn and Harrison [32] using the procedure used by Cook [33]. This index ranges from −1 to +1; based on the same sampling effort, positive values indicate that collections of small patches contain more species than fewer larger patches, while negative values indicate the opposite. To assess the relative importance of the landscape spatial context on these results, we compared these species-area curves with those found in three neighboring fragmented tropical landscapes located in the Los Tuxtlas rainforest, Mexico, but with higher deforestation levels (24%, 11% and 4% of remaining forest cover). The species-area curves in this case were generated using the same protocol described above, based on a vegetation data set that was collected by VAR using the same sampling methodology and sampling effort as in the Lacandona rainforest [7], [8]. The only difference was that Arroyo-Rodríguez et al. [7], [8] included not only trees and palms, but also lianas. Thus, we excluded lianas from the original data set of VAR to make the results comparable with the present study.

## Results

We recorded 6222 trees from 55 families, 144 genera, and 228 species (including 24 morphospecies) in a total sampled area of 3 ha (Table S1). On average (± SD), we recorded 28±5 families, 50±9 genera, and 59±12 species per site (Table 1). Stem density averaged 207±34 stems/0.1 ha (Table 2). We also recorded 15±6 dead trees, and 4±4 logged trees per site. Basal area averaged 3.4±0.87 m^2^ per site. Finally, the evenness factor averaged 0.41±0.13 (Table 2).

**Table 2:** Vegetation structure in 26 forest patches (FP) and 4 reference sites within a continuous forest (CF) sampled in the fragmented Lacandon rainforest, Chiapas, Mexico.

Sites	Vegetation characteristics				
	# stems	# dead trees	# logged trees	BA (m^2^)	*EF*
FP1	260	18	15	3.00	0.39
FP2	168	13	4	3.67	0.35
FP3	209	16	0	3.93	0.37
FP4	197	12	4	2.57	0.42
FP5	228	21	3	3.40	0.47
FP6	185	13	10	2.79	0.50
FP7	193	17	5	4.35	0.54
FP8	214	22	2	3.84	0.56
FP9	140	7	7	3.36	0.17
FP10	231	9	5	2.70	0.50
FP11	216	28	7	2.67	0.55
FP12	192	19	3	4.31	0.39
FP13	199	12	5	4.15	0.52
FP14	174	10	2	4.84	0.29
FP15	237	24	0	1.98	0.54
FP16	206	9	3	2.65	0.33
FP17	165	13	0	3.57	0.41
FP18	151	10	4	2.95	0.63
FP19	239	18	0	2.48	0.27
FP20	209	11	6	3.54	0.47
FP21	154	8	7	2.57	0.59
FP22	229	28	10	3.85	0.41
FP23	238	10	1	5.11	0.36
FP24	255	8	1	3.32	0.23
FP25	233	17	1	3.02	0.44
FP26	279	14	0	2.29	0.13
CF1	198	11	1	2.14	0.39
CF2	228	11	1	3.40	0.18
CF3	212	17	0	5.54	0.50
CF4	183	8	0	3.36	0.25

The number of stems, dead trees, and logged trees are indicated, as well as basal area (BA, m^2^) and the evenness factor (*EF*) estimated for ten 50×2-m transects (0.1 ha) per site. Sites are arranged in order of increasing size (see site sizes in Table 1). *EF* represents the proportion of dominant species in the community.

The four largest families in the region were Fabaceae (27 species), Rubiaceae (13), Malvaceae (11), and Moraceae (10), which combined accounted for 30% of all species recorded (Table S1). In contrast, 25 families (46%) were represented by only one species. Acanthaceae, Asteraceae, Cannabaceae, Malpighiaceae, and Nyctaginaceae (9% of all families) were recorded in only one forest patch, and Passifloraceae (2%) was exclusive to a single continuous forest area. The most diverse genus was *Piper* (Piperaceae) with six species, whereas 106 genera (75%) were represented by a single species (Table S1).

All species were native to the region. Eleven out of the 204 species (5%) are classified within a risk category by the Mexican government: *Bactris major*, listed as species subjected to special protection; *Astronium graveolens*, *Bravaisia integerrima*, *Calophyllum brasiliense*, *Cryosophila stauracantha*, *Geonoma interrupta*, *Guatteria anomala*, *Magnolia mexicana*, and *Spondias radlkoferi*, listed as threatened species; and *Ormosia isthmensis* and *Vatairea lundellii*, listed as endangered species (Table S1). All these species were recorded in forest patches (Table S1). Furthermore, we reported for the first time the occurrence of *Ouratea crassinervia* Engl. (Ochnaceae) for the Mexican flora (see online Appendix S1). This species was recorded in two areas within the continuous forest and in two forest patches (Table S1).

**Figure 2:**
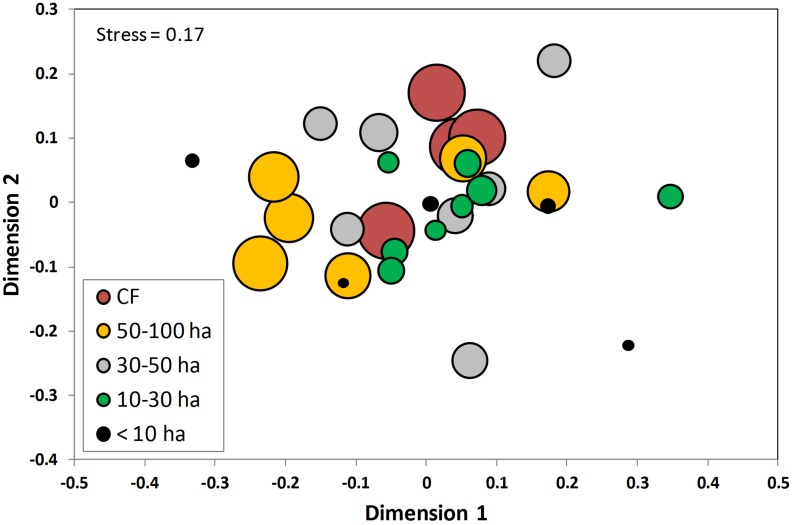
Non-metric multidimensional scaling ordination of tree species across each site sampled in the fragmented Lacandon rainforest, Mexico. Dot size is directly proportional to site size. CF = continuous forest.

Analyzing the full set of sites (n = 30), there were no significant relationships between patch size and vegetation attributes: rarefied number of families (*R^2^* = 0.002, *P* = 0.80), genera (*R^2^* = 0.022, *P* = 0.43), and species (*R^2^* = 0.034, *P* = 0.33), density of stems (*R^2^* = 0.016, *P* = 0.50) and dead trees (*R^2^* = 0.041, *P* = 0.28), basal area (*R^2^* = 0.001, *P* = 0.91) and evenness factor (*R^2^* = 0.045, *P* = 0.26). Only the density of logged trees was negatively correlated with patch size (*R^2^* = 0.207, *P* = 0.01). Based on the presence and absence of species within the sites (Jaccard index), we found that on average they shared 27±9% species, indicating a high species turnover among sites. Considering the abundance of individuals, the NMDS ordination showed a strong variation in species composition among sites, and this variation was unrelated to patch size (Figure 2), as MANOVA did not detect differences among size classes in any of the ordination dimensions generated by the NMDS analysis (*F*_8,48_ = 0.896, *P* = 0.53). Furthermore, the Mantel tests revealed a non-significant correlation between floristic similarity and differences in size among sites (*r* = −0.03, *P* = 0.5), and a significant but very weak correlation between floristic similarity and geographic distances among sites (*r* = 0.15, *P* = 0.004).

**Figure 3:**
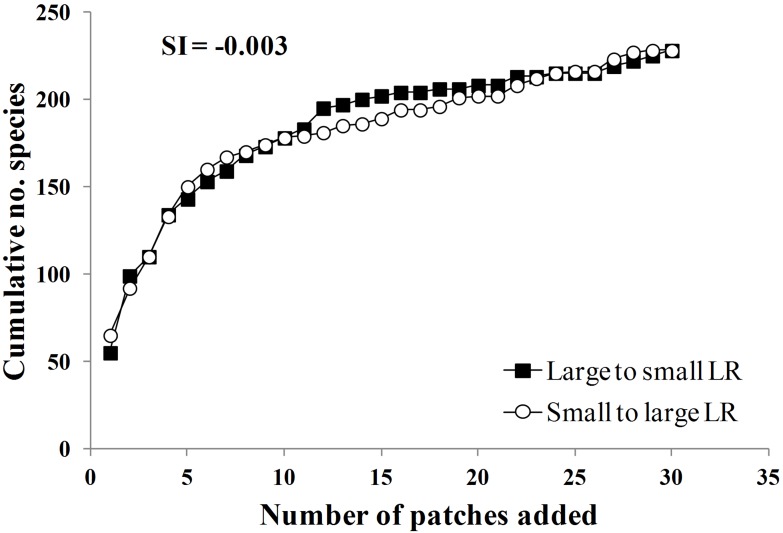
Cumulative species-area curves for Lacandon rainforest, Mexico. Cumulative number of species sampled in 0.1 per patch versus number of patches added. Patches were added from small to large or large to small, respectively, and then corresponding cumulative species counts were obtained. The value of Saturation Index is indicated.

**Figure 4:**
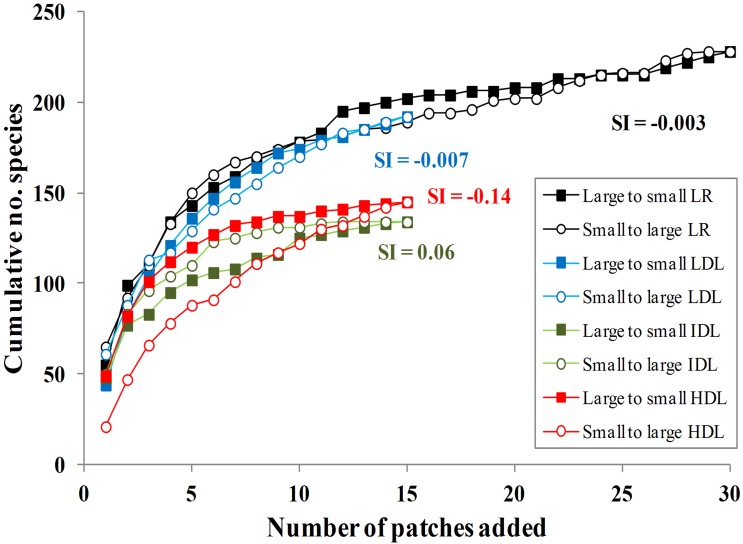
Cumulative number of tree species sampled in 0.1 ha per patch versus the number of patches added in four landscapes with different degrees of deforestation. In the Lacandon rainforest (LR; with approximately 40% of remaining forest cover) we sampled 30 forest patches (present study). Using the same sampling methodology and sampling effort as in LR, in the Los Tuxtlas rainforest, Mexico we sampled 15 patches in three landscapes (see Arroyo-Rodríguez et al. [7]): LDL (lowest deforestation, 24%); IDL (intermediate deforestation, 11%); and HDL (highest deforestation, 4%). In all cases, patches were added from large to small (squares) or small to large (circles), and then, corresponding cumulative species counts were obtained. The Saturation Index (SI) indicated that only in the landscape with highest deforestation level (HDL) larger patches accumulated more species than smaller ones with the same sample intensity.

The cumulative species-area curves displayed similar patterns of species accumulation in both treatments, either from small to large and large to small (Figure 3). The saturation index (SI = −0.003) indicated that the resulting species-area curves were indistinguishable, regardless of whether small or large patches were added first. This pattern was almost identical to that observed in the landscape with lower deforestation level (24% of forest cover) at Los Tuxtlas, Mexico; however, in this region, the landscape with highest deforestation level (4% of forest cover) showed that collections of small patches contained a lower number of species than fewer larger patches (SI = −0.14; Figure 4), indicating that the relative contribution of small forest patches to regional diversity declines in landscapes with lower forest cover.

## Discussion

It is clear from our study that small forest patches in the fragmented Lacandon rainforest can still harbor original levels of tree species diversity and forest structure, as if they were part of a continuous forest. These patches maintained diverse communities of native tree species, including endangered taxa (see Table S1) and a new record for the Mexican flora (see additional and recent new records for the region in [34]–[36]). Surprisingly, patch size was a poor indicator of tree species diversity and forest structure, indicating that even the smallest forest patches were highly similar to continuous forest sites in terms of total basal area, community evenness, stem density and number of species, genera, and families. Also, the proportion of pioneer species (i.e., early-colonizer species) within each site – an indicative of forest disturbance [10]–[13] – was independent of site size (see online Figure S1 in Appendix S2). Finally, each patch conserved a particular floristic composition (i.e., high β-diversity among sites), which was determined neither by differences in patch size nor by geographical distance. Thus, the preservation of both small and large patches is necessary for conserving regional plant species diversity.

Our findings contrast with those found in other fragmented tropical landscapes with higher deforestation levels ([7]; Figure 4) and longer history of contemporary human activity (e.g., [10]). For example, several studies from South American fragmented rainforests indicate that many tree species can disappear from small forest patches surrounded by pastures or sugarcane fields [10]–[13]. This local species extirpation has been mainly related to edge effects, i.e., to altered biotic and abiotic conditions near forest edges [10]–[13]. Several studies have reported that a number of disturbance-adapted native species (e.g., short- and long-lived light-demanding species) can dominate plant communities in fragmented forests with pronounced edge effects [11], [37]. This process can reduce community evenness and increase floristic homogenization (i.e., reduced β-diversity) across multiple spatial scales [13], [38].

These contrasting findings may be related to the fact that species maintenance in forest patches largely depends on the landscape context [6]. For example, several studies have demonstrated that species extirpation in smaller forest patches is only evident below a given threshold of landscape forest cover (e.g., <11% [7]; <10–30% [39]; <20% [40]), and our study landscape has a notably greater remaining forest cover (ca. 40%). Additionally, human population density in the region is amongst the lowest in Mexico (4.8 people/km^2^ [41]), and regional land use is dominated by familiar cattle ranches and *milpas*, a sort of small-scale agriculture for subsistence and local commerce that is known to have a lower impact if compared with agribusiness activities typical from other tropical countries, such as Brazil [2], [6]. This reinforces the context-dependency in the determination of how much biodiversity a human-modified landscape can harbour [6] and claims for the attention to the socioecological context where tropical landscapes rely.

The ability of small forest patches to maintain biodiversity also depends on the landscape matrix [6], [22], [42], as it determines the severity of edge effects [43]–[45]. Edge effects, or the environmental changes that occur near forest edges, can increase tree mortality rates in forest patches, particularly when the landscape is dominated by agricultural matrices of intensive uses (e.g., large pastures and monocultures [9], [43]). However, in fragmented landscapes with a relatively high forest cover and a heterogeneous matrix, such as the Lacandon region, we may expect such environmental changes to be attenuated [43], [44]. Thus, these processes, in conjunction with the fact that many tropical trees are long-lived [46], can contribute to prevent tree mortality and maintain the overall patterns of forest structure even in the smallest patches, at least in the short-term. Certainly, further studies are needed to monitor tree mortality and assess the long-term maintenance of species diversity within these patches.

### Management and Conservation Implications

Our findings indicate that a half century of human occupancy in the region has not resulted in significant changes in tree assemblages within small forest patches. This is crucial for providing suitable habitat required by many closed-canopy forest animal groups, and the preservation of the ecological processes in which they are involved (e.g., seed dispersal, seed predation, and pollination), and that are necessary for forest regeneration. The importance of small patches goes beyond their values as species reservoirs, as they can also act as buffer zones in which some management practices can be allowed, reducing the pressure over the largest patches. Even the smallest patches can contribute to landscape connectivity, because a set of several small patches distributed across the landscape may serve as stepping stones, increasing the connectivity among patches and enhancing inter-patch animal and plant dispersal [7], [21], [31].

Unfortunately, the long-term persistence of these patches is uncertain, as the focus of national and international conservation efforts has been to safeguard the largest forest remnants. In particular, economic mechanisms and incentives such as the payments for ecosystem services (PES) have been increasingly implemented as a promissory conservation approach in this and many other Mexican forests [47]. Yet in the Lacandon region, the recently created ‘Programa Especial Selva Lacandona 2013’ [48] only offers economic benefits (ca. 77 US$/ha/year) to landowners and local communities that are preserving >25 ha of forest. Below this area, local people cannot apply to this program, limiting the economic and ecological value of small forest patches. This situation will obviously increase the anthropogenic pressures on these small, but highly valuable forest patches.

Nonetheless, we are not arguing that funding and other conservation efforts of larger forest patches should be re-directed towards smaller ones. The great value of large blocks of original ecosystem for conservation is incontrovertible, but what is questionable is the exclusion of smaller forest patches from conservation initiatives. In the tropical rainforest of Mexico, either in the Gulf of Mexico [7], [8] or in the Lacandon region (present study), not protecting smaller forest patches is a waste of biodiversity. We stress that, supporting a land-sharing approach, future conservation and management initiatives should consider the importance of protecting both small- and large-sized forest patches.

## Supporting Information

Table S1 **Check list of the tree species recorded in 26 forest patches and 4 reference sites within a continuous forest located in the Lacandon rainforest, Chiapas, Mexico.**
(DOCX)

Appendix S1 **Novelty for the Lacandon rainforest flora, Chiapas, Mexico.**
(DOCX)

Appendix S2 **Proportion of pioneer (i.e., early-colonizer) and persistent (i.e., late-successional and old-growth forest) plant species within different-sized forest patches and continuous forest sites in the Lacandon rainforest, Mexico.**
(DOCX)

## References

[1] Sanderson EW, Jaiteh M, Levy MA, Redford KH, Wannebo AV, et al (2002) The human footprint and the last of the wild. Bioscience 52: 891–904.

[2] Laurance WF, Sayer J, Cassman KG (2014) Agricultural expansion and its impacts on tropical nature. Trends Ecol Evol 29: 107–116. 24388286 10.1016/j.tree.2013.12.001

[3] Lindenmayer D, Hobbs RJ, Montague-Drake R, Alexandra J, Bennett A, et al (2008) A checklist for ecological management of landscapes for conservation. Ecol Lett 11: 78–91. 17927771 10.1111/j.1461-0248.2007.01114.x

[4] Gardner TA, Barlow J, Chazdon R, Ewers RM, Harvey CA, et al (2009) Prospects for tropical forest biodiversity in a human-modified world. Ecol Lett 12: 561–582. 19504750 10.1111/j.1461-0248.2009.01294.x

[5] Chazdon RL, Harvey CA, Komar O, Griffith DM, Ferguson BG, et al (2009) Beyond reserves: a research agenda for conserving biodiversity in human-modified tropical landscapes. Biotropica 41: 142–153.

[6] Melo FPL, Arroyo-Rodríguez V, Fahrig L, Martínez-Ramos M, Tabarelli M (2013) On the hope for biodiversity-friendly tropical landscapes. Trends Ecol Evol 28: 461–468. 10.1016/j.tree.2013.01.001 23375444

[7] Arroyo-Rodríguez V, Pineda E, Escobar F, Benítez-Malvido J (2009) Value of small patches in the conservation of plant-species diversity in highly fragmented rainforest. Conserv Biol 23: 729–739. 19040651 10.1111/j.1523-1739.2008.01120.x

[8] Arroyo-Rodríguez V, Cavender-Bares J, Escobar F, Melo FPL, Tabarelli M, et al (2012) Maintenance of tree phylogenetic diversity in a highly fragmented rain forest. J Ecol 100: 702–711.

[9] Laurance WF, Delamônica P, Laurance SG, Vasconcelos HL, Lovejoy TE (2000) Rainforest fragmentation kills big trees. Nature 404: 836. 10786782 10.1038/35009032

[10] Silva JMC, Tabarelli M (2000) Tree species impoverishment and the future flora of the Atlantic forest of northeast Brazil. Nature 404: 72–74. 10716443 10.1038/35003563

[11] Laurance WF, Nascimento HEM, Laurance SG, Andrade A, Ribeiro JELS, et al (2006) Rapid decay of tree-community composition in Amazonian forest fragments. Proc Natl Acad Sci USA 103: 19010–19014. 17148598 10.1073/pnas.0609048103 PMC1682011

[12] Santos BA, Peres CA, Oliveira MA, Grillo A, Alves-Costa CP, et al (2008) Drastic erosion in functional attributes of tree assemblages in Atlantic forest fragments of northeastern Brazil. Biol Conserv 141: 249–260.

[13] Tabarelli M, Peres CA, Melo FPL (2012) The ‘few winners and many losers' paradigm revisited: emerging prospects for tropical forest biodiversity. Biol Conserv 155: 136–140.

[14] Turner IM, Corlett RT (1996) The conservation value of small, isolated fragments of lowland tropical rain forest. Trends Ecol Evol 11: 330–333. 21237864 10.1016/0169-5347(96)10046-x

[15] Myers N, Mittermeier RA, Mittermeier CG, da Fonseca GAB, Kent J (2000) Biodiversity hotspots for conservation priorities. Nature 403: 853–858. 10706275 10.1038/35002501

[16] Martínez E, Ramos CH, Chiang F (1994) Lista florística de la Lacandona, Chiapas. Bol Soc Bot Mex 54: 99–177.

[17] Ibarra-Manríquez G, Martínez-Ramos M (2002) Landscape variation of liana communities in a Neotropical rain forest. Plant Ecol 160: 91–112.

[18] van Breugel M, Martínez-Ramos M, Bongers F (2006) Community dynamics during early secondary succession in Mexican tropical rain forests. J Trop Ecol 22: 663–674.

[19] Fahrig L (2003) Effects of habitat fragmentation on biodiversity. Annu Rev Ecol Evol Syst 34: 487–515.

[20] Pardini R, Bueno AA, Gardner TA, Prado PI, Metzger JP (2010) Beyond the fragmentation threshold hypothesis: regime shifts in biodiversity across fragmented landscapes. PLoS ONE 5: e13666. 21060870 10.1371/journal.pone.0013666 PMC2965145

[21] Ewers RM, Didham RK (2006) Confounding factors in the detection of species responses to habitat fragmentation. Biol Rev 81: 117–142. 16318651 10.1017/S1464793105006949

[22] Fahrig L, Baudry J, Brotons L, Burel FG, Crist TO, et al (2011) Functional landscape heterogeneity and animal biodiversity in agricultural landscapes. Ecol Lett 14: 101–112. 21087380 10.1111/j.1461-0248.2010.01559.x

[23] Garmendia A, Arroyo-Rodríguez V, Estrada A, Naranjo E, Stoner KE (2013) Landscape and patch attributes impacting medium- and large-sized terrestrial mammals in a fragmented rain forest. J Trop Ecol 29: 331–344.

[24] Martínez E (2003) Marquéz de Comillas. In: Lichtinger V, Enkerlin E, Enríquez C, García P, editors. La Deforestación en 24 Regiones PRODERS. Mexico City: Secretaría de Medio Ambiente y Recursos Naturales, 124–131.

[25] Aguilar LF, Mora CS (1992) Colonización y deterioro de la selva Lacandona. Rev Geogr 116: 67–84.

[26] Gentry AH (1982) Patterns of Neotropical plant species diversity. In: Hecht MK, Wallace B, Prance GT, editors. Evolutionary Biology. New York: Plenum Press, 1–84.

[27] Chao A, Jost L (2012) Coverage-based rarefaction and extrapolation: standardizing samples by completeness rather than size. Ecology 93: 2533–2547. 23431585 10.1890/11-1952.1

[28] Chao A, Gotelli NJ, Hsieh TC, Sander EL, Ma KH, et al (2014) Rarefaction and extrapolation with Hill numbers: a framework for sampling and estimation in species diversity studies. Ecol Monogr (doi:10.1890/13-0133.1)

[29] Hsieh TC, Ma KH, Chao A (2013) iNEXT online: interpolation and extrapolation (Version 1.3.0) Software. Available: http://chao.stat.nthu.edu.tw/blog/software-download/.

[30] Jost L (2010) The relation between evenness and diversity. Diversity 2: 207–232.

[31] Fischer J, Lindenmayer DB (2002) Small patches can be valuable for biodiversity conservation: two case studies on birds in southeastern Australia. Biol Conserv 106: 129–136.

[32] Quinn JF, Harrison SP (1988) Effects of habitat fragmentation and isolation on species richness: evidence from biogeographic patterns. Oecologia 75: 132–140. 28311846 10.1007/BF00378826

[33] Cook RR (1995) The relationship between nested subsets, habitat subdivision, and species diversity. Oecologia 101: 204–210. 28306792 10.1007/BF00317285

[34] Ibarra-Manríquez G, Cornejo-Tenorio G, González-Castañeda N, Piedra-Malagón EM, Luna A (2012) El género *Ficus* L. (Moraceae) en México. Bot Sci 90: 389–452.

[35] Santos BA, Lombera R, Benítez-Malvido J (2009) New records of *Heliconia* (Heliconiaceae) for the region of Chajul, Southern Mexico, and their potential use in biodiversity-friendly cropping systems. Rev Mex Biodivers 80: 857–860.

[36] Schutzman B, Vovides AP (1998) A new *Zamia* (Zamiaceae, Cycadales) from Eastern Chiapas, Mexico. Novon 8: 441–446.

[37] Santos GGA, Santos BA, Nascimento HEM, Tabarelli M (2012) Contrasting demographic structure of short- and long-lived pioneer tree species on Amazonian forest edges. Biotropica 44: 771–778.

[38] Lôbo D, Leão T, Melo FPL, Santos AMM, Tabarelli M (2011) Forest fragmentation drives Atlantic forest of northeastern Brazil to biotic homogenization. Divers Distrib 17: 287–296.

[39] Andrén H (1994) Effects of habitat fragmentation on birds and mammals in landscapes with different proportions of suitable habitat: a review. Oikos 71: 355–366.

[40] Fahrig L (1998) When does fragmentation of breeding habitat affect population survival? Ecol Model 105: 273–292.

[41] SEDESOL (2014) Secretaría de Desarrollo Social. Microrregiones-Catálogo de localidades. Available: http://www.microrregiones.gob.mx/catloc/LocdeMun.aspx?tipo=clave&campo=loc&ent=07&mun=116 Accessed 10 January 2014.

[42] Perfecto I, Vandermeer J. Wright A (2009) Nature’s matrix: linking agriculture, conservation and food sovereignty. London: Earthscan.

[43] Mesquita R, Delamonica P, Laurance WF (1999) Effects of surrounding vegetation on edge-related tree mortality in Amazonian forest fragments. Biol Conserv 91: 129–134.

[44] Murcia C (1995) Edge effects in fragmented forests: implications for conservation. Trends Ecol Evol 10: 58–62. 21236953 10.1016/S0169-5347(00)88977-6

[45] Pinto SRR, Mendes G, Santos AMM, Dantas M, Tabarelli M, et al (2010) Landscape attributes drive complex spatial microclimate configuration of Brazilian Atlantic forest fragments. Trop Conserv Sci 3: 399–402.

[46] Martínez-Ramos M, Álvarez-Buylla ER (1998) How old are tropical rain forest trees? Trends Plant Sci 3: 400–405.

[47] CONAFOR (2010) Coordinación General de Producción y Productividad. Gerencia de Servicios Ambientales del Bosque. Mexico City: Comisión Nacional Forestal.

[48] CONAFOR (2013). Programa Especial Selva Lacandona 2013. Coordinación General de Producción y Productividad. Gerencia de Servicios Ambientales del Bosque. Mexico City: Comisión Nacional Forestal.

