# Supplementary material for: Conserving Tropical Tree Diversity and Forest Structure: The Value of Small Rainforest Patches in Moderately-Managed Landscapes
Source: PLoS One. 2014 Jun 5;9(6):e98931. doi: 10.1371/journal.pone.0098931 (PMC4047051; doi:10.1371/journal.pone.0098931)
Supplement: Table S1 — Check list of the tree species recorded in 26 forest patches and 4 reference sites within a continuous forest located in the Lacandon rainforest, Chiapas, Mexico. (DOCX) [file pone.0098931.s001.docx]

**Table S1 Check list of the tree species recorded in 26 forest patches and 4 reference sites within a continuous forest located in the Lacandon rainforest, Chiapas, Mexico**. Plant names followed the Missouri Botanical Garden electronic database (Tropicos) available at http://www.tropicos.org. Abundance and proportion of occupied forest patches (FP) and continuous forest (CF) sites by each species are also indicated. Eleven out of the 204 identified species (5%) are classified within a risk category by the Mexican government (indicated with an asterisk, * [1]).

| **Family** | **Species** | **CF** | **FP** | **Total** |
| --- | --- | --- | --- | --- |
| Acanthaceae | *Bravaisia integerrima* (Spreng.) Standl.* | - | 7 (0.04) | 7 |
| Anacardiaceae | *Astronium graveolens* Jacq.* | 1 (0.25) | 16 (0.31) | 17 |
|  | *Mosquitoxylum jamaicense* Krug & Urb. | - | 1 (0.04) | 1 |
|  | *Spondias mombin* L. | 1 (0.25) | 8 (0.15) | 9 |
|  | *Spondias radlkoferi* Donn. Sm.* | 5 (0.75) | 64 (0.69) | 69 |
| Annonaceae | *Annona scleroderma* Saff. | 2 (0.25) | 2 (0.08) | 4 |
|  | *Cymbopetalum mayanum* Lundell | 9 (0.75) | 41 (0.69) | 50 |
|  | *Guatteria anomala* R.E. Fr.* | - | 2 (0.04) | 2 |
|  | *Guatteria* sp. | 2 (0.25) | 14 (0.27) | 16 |
|  | *Mosannona depressa* (Baill.) Chatrou | - | 1 (0.04) | 1 |
|  | *Xylopia frutescens* Aubl. | 1 (0.25) | 35 (0.38) | 36 |
| Apocynaceae | *Stemmadenia donnell-smithii* (Rose) Woodson | 2 (0.25) | - | 2 |
|  | *Stemmadenia litoralis* (Kunth) L. Allorge | 5 (0.50) | 23 (0.42) | 28 |
|  | *Tabernaemontana alba* Mill. | - | 28 (.50) | 28 |
|  | *Thevetia ahouai* (L.) A. DC. | 3 (0.50) | 17 (0.42) | 20 |
| Araliaceae | *Dendropanax arboreus* (L.) Decne. & Planch. | - | 26 (0.54) | 26 |
|  | *Oreopanax peltatus* Linden | - | 14 (0.15) | 14 |
|  | *Schefflera morototoni* (Aubl.) Maguire, Steyerm. & Frodin | 3 (0.50) | 1 (0.04) | 4 |
| Arecaceae | *Attalea rostrata* Oerst. | - | 2 (0.08) | 2 |
|  | *Bactris major* Jacq.* | - | 63 (0.15) | 63 |
|  | *Bactris mexicana* Mart. | 14 (1.00) | 145 (0.88) | 159 |
|  | *Chamaedorea tepejilote* Liebm. | 4 (0.50) | 39 (0.31) | 43 |
|  | *Cryosophila stauracantha* (Heynh.) R. Evans* | 8 (0.50) | 16 (0.08) | 24 |
|  | *Geonoma interrupta* (Ruiz & Pav.) Mart.* | 17 (1.00) | 40 (0.50) | 57 |
|  | *Sabal mauritiiformis* (H. Karst.) Griseb. & H. Wendl. | - | 9 (0.19) | 9 |
|  | *Sabal mexicana* Mart. | - | 3 (0.08) | 3 |
| Asparagaceae | *Dracaena americana* Donn. Sm. | - | 6 (0.19) | 6 |
| Asteraceae | *Critonia morifolia* (Mill.) R.M. King & H. Rob. | - | 1 (0.04) | 1 |
| Bignoniaceae | *Amphitecna apiculata* A.H. Gentry | 5 (1.00) | 55 (0.65) | 60 |
|  | *Handroanthus guayacan* (Seem.) S. O. Grose | - | 1 (0.04) | 1 |
|  | *Tabebuia rosea* (Bertol.) A. DC. | - | 1 (0.04) | 1 |
| Boraginaceae | *Cordia alliodora* (Ruiz & Pav.) Oken | - | 3 (0.04) | 3 |
|  | *Cordia bicolor* A. DC. | 1 (0.25) | 29 (0.54) | 30 |
|  | *Cordia diversifolia* Pav. ex DC. | - | 6 (0.15) | 6 |
|  | *Cordia* sp. | - | 1 (0.04) | 1 |
| Burseraceae | *Bursera simaruba* (L.) Sarg. | 1 (0.25) | 37 (0.50) | 38 |
|  | *Protium confusum* (Rose) Pittier | - | 80 (0.35) | 79 |
|  | *Protium multiramiflorum* Lundell | 27 (1.00) | 130 (0.85) | 157 |
| Calophyllaceae | *Calophyllum brasiliense* Cambess.* | 3 (0.50) | 25 (0.35) | 28 |
| Cannabaceae | *Trema micrantha* (L.) Blume | - | 1 (0.04) | 1 |
| Capparaceae | *Capparis quiriguensis* Standl. | - | 7 (0.08) | 7 |
| Caricaceae | *Carica papaya* L. | - | 1 (0.04) | 1 |
|  | *Jacaratia dolichaula* (Donn. Sm.) Woodson | 4 (0.75) | 29 (0.54) | 33 |
| Celastraceae | *Crossopetalum parviflorum* (Hemsl.) Lundell | - | 1 (0.04) | 1 |
|  | *Crossopetalum* sp. 1 | 16 (0.75) | 49 (0.54) | 65 |
|  | *Crossopetalum* sp. 2 | 4 (0.75) | 5 (0.15) | 9 |
| Chrysobalanaceae | *Hirtella americana* L. | 7 (0.50) | 37 (0.38) | 44 |
|  | *Hirtella racemosa* Lam. | 13 (0.75) | 38 (0.42) | 51 |
|  | *Licania hypoleuca* Benth. | 4 (0.25) | 123 (0.62) | 127 |
|  | *Licania platypus* (Hemsl.) Fritsch | 3 (0.50) | 16 (0.31) | 19 |
| Clethraceae | *Clethra occidentalis* (L.) Kuntze | 2 (0.25) | 8 (0.08) | 10 |
| Clusiaceae | *Garcinia intermedia* (Pittier) Hammel | 1 (0.25) | 18 (0.23) | 19 |
| Combretaceae | *Terminalia amazonia* (J.F. Gmel.) Exell | 5 (0.50) | 68 (0.54) | 73 |
| Elaeocarpaceae | *Sloanea tuerckheimii* Donn. Sm. | - | 12 (0.23) | 12 |
| Erythroxylaceae | *Erythroxylum macrophyllum* Cav. | 2 (0.25) | 8 (0.15) | 10 |
| Euphorbiaceae | *Acalypha diversifolia* Jacq. | 4 (0.75) | 50 (0.35) | 54 |
|  | *Alchornea latifolia* Sw. | 2 (0.25) | 28 (0.38) | 30 |
|  | *Croton draco* Schltdl. & Cham. | 1 (0.25) | - | 1 |
|  | *Croton schiedeanus* Schltdl. | 4 (0.50) | 81 (0.73) | 85 |
|  | *Croton* sp. | - | 2 (0.08) | 2 |
|  | *Sapium lateriflorum* Hemsl. | - | 22 (0.31) | 22 |
|  | *Pleradenophora tuerckheimiana* (Pax & K. Hoffm.) A. L. Melo & Esser | 1 (0.25) | 8 (0.15) | 9 |
| Fabaceae | *Albizia leucocalyx* (Britton & Rose) L. Rico | 2 (0.50) | 15 (0.27) | 17 |
|  | *Cojoba arborea* (L.) Britton & Rose | - | 8 (0.19) | 8 |
|  | *Cojoba graciliflora* (S.F. Blake) Britton & Rose | - | 1 (0.04) | 1 |
|  | *Dalbergia glabra* (Mill.) Standl. | 1 (0.25) | 8 (0.23) | 9 |
|  | *Dalbergia glomerata* Hemsl. | 1 (0.25) | 20 (0.23) | 21 |
|  | *Dialium guianense* (Aubl.) Sandwith | 38 (1.00) | 162 (0.88) | 200 |
|  | *Erythrina folkersii* Krukoff & Moldenke | 1 (0.25) | 9 (0.27) | 10 |
|  | *Hymenaea courbaril* L. | 1 (0.25) | - | 1 |
|  | *Indigofera suffruticosa* Mill. | - | 9 (0.23) | 9 |
|  | *Inga pavoniana* G. Don | 3 (0.75) | 46 (0.69) | 49 |
|  | *Inga punctata* Willd. | - | 13 (0.31) | 13 |
|  | *Inga thibaudiana* DC. | 3 (0.25) | 29 (0.31) | 32 |
|  | *Lonchocarpus cruentus* Lundell | - | 29 (0.46) | 29 |
|  | *Lonchocarpus guatemalensis* Benth. | 2 (0.25) | 8 (0.23) | 10 |
|  | *Lonchocarpus rugosus* Benth. | - | 4 (0.12) | 4 |
|  | *Mariosousa usumacintensis* (Lundell) Seigler & Ebinger | 1 (0.25) | 8 (0.27) | 9 |
|  | *Ormosia isthmensis* Standl.* | 1 (0.25) | 4 (0.12) | 5 |
|  | *Pithecellobium macrandrium* Donn. Sm. | 2 (0.50) | 24 (0.46) | 26 |
|  | *Platymiscium dimorphandrum* Donn. Sm. | 2 (0.25) | 6 (0.19) | 8 |
|  | *Pterocarpus rohrii* Vahl | 3 (0.75) | 8 (0.27) | 11 |
|  | *Schizolobium parahyba* (Vell.) S.F. Blake | 1 (0.25) | 71 (0.50) | 72 |
|  | *Senegalia polyphylla* (DC.) Britton | - | 1 (0.04) | 1 |
|  | *Swartzia guatemalensis* (Donn. Sm.) Pittier | 3 (0.25) | 6 (0.15) | 9 |
|  | *Vachellia cornigera* (L.) Seigler & Ebinger | - | 47 (0.38) | 47 |
|  | *Vachellia mayana* (Lundell) Seigler & Ebinger | - | 10 (0.19) | 10 |
|  | *Vatairea lundellii* (Standl.) Killip ex Record* | 3 (0.50) | 38 (0.58) | 41 |
|  | *Zygia peckii* (B.L. Rob.) Britton & Rose | - | 35 (0.38) | 35 |
| Hypericaceae | *Vismia camparaguey* Sprague & L. Riley | - | 8 (0.12) | 8 |
| Lacistemataceae | *Lacistema aggregatum* (P.J. Bergius) Rusby | 9 (0.25) | 79 (0.65) | 88 |
| Lauraceae | *Licaria caudata* (Lundell) Kosterm. | 2 (0.50) | 10 (0.23) | 12 |
|  | *Licaria excelsa* Kosterm. | 1 (0.25) | 17 (0.38) | 18 |
|  | *Nectandra ambigens* (S.F. Blake) C.K. Allen | - | 2 (0.08) | 2 |
|  | *Nectandra belizensis* (Lundell) C.K. Allen | 3 (0.50) | 14 (0.38) | 17 |
|  | *Nectandra reticulata* (Ruiz & Pav.) Mez | 4 (0.75) | 41 (0.73) | 45 |
|  | *Nectandra salicifolia* (Kunth) Nees | 12 (0.75) | 61 (0.65) | 73 |
|  | *Ocotea* sp. | 2 (0.50) | 16 (0.35) | 18 |
| Magnoliaceae | *Magnolia mexicana* DC.* | 2 (0.25) | 6 (0.08) | 8 |
| Malpighiaceae | *Byrsonima crassifolia* (L.) Kunth | - | 1 (0.04) | 1 |
| Malvaceae | *Ceiba pentandra* (L.) Gaertn. | - | 1 (0.04) | 1 |
|  | *Guazuma ulmifolia* Lam. | - | 2 (0.08) | 2 |
|  | *Hampea stipitata* S. Watson | 1 (0.25) | 13 (0.23) | 14 |
|  | *Luehea seemannii* Triana & Planch. | - | 1 (0.04) | 1 |
|  | *Luehea speciosa* Willd. | 1 (0.25) | 45 (0.31) | 46 |
|  | *Mortoniodendron sulcatum* Lundell | 1 (0.25) | - | 1 |
|  | *Pachira aquatica* Aubl. | 4 (0.50) | 23 (0.50) | 27 |
|  | *Quararibea funebris* (La Llave) Vischer | 3 (0.50) | 26 (0.19) | 29 |
|  | *Quararibea yunckeri* Standl. | 11 (0.75) | 56 (0.54) | 67 |
|  | *Theobroma cacao* L. | 1 (0.25) | 11 (0.04) | 12 |
|  | *Trichospermum mexicanum* (DC.) Baill. | - | 30 (0.31) | 30 |
| Melastomataceae | *Bellucia pentamera* Naudin | - | 4 (0.12) | 4 |
|  | *Conostegia xalapensis* (Bonpl.) D. Don ex DC. | - | 2 (0.08) | 2 |
|  | *Miconia argentea* (Sw.) DC. | 15 (0.75) | 96 (0.58) | 111 |
|  | *Miconia fulvostellata* L.O. Williams | - | 1 (0.04) | 1 |
|  | *Miconia trinervia* (Sw.) D. Don ex Loudon | - | 24 (0.31) | 24 |
|  | *Mouriri gleasoniana* Standl. | - | 7 (0.19) | 7 |
|  | *Mouriri myrtilloides* (Sw.) Poir. | 5 (0.75) | 12 (0.27) | 17 |
| Meliaceae | *Guarea glabra* Vahl | 116 (1.00) | 195 (0.77) | 311 |
|  | *Guarea grandifolia* DC. | 1 (0.25) | 16 (0.42) | 17 |
|  | *Swietenia macrophylla* King | - | 19 (0.15) | 19 |
|  | *Trichilia erythrocarpa* Lundell | 5 (0.50) | 10 (0.15) | 15 |
| Monimiaceae | *Mollinedia viridiflora* Tul. | 4 (0.50) | 51 (0.46) | 55 |
| Moraceae | *Brosimum alicastrum* Sw. | 9 (1.00) | 104 (0.73) | 113 |
|  | *Brosimum guianense* (Aubl.) Huber | 16 (0.75) | 49 (0.65) | 65 |
|  | *Castilla elastica* Sessé | 9 (0.75) | 126 (0.81) | 135 |
|  | *Clarisia biflora* Ruiz & Pav. | - | 29 (0.42) | 29 |
|  | *Ficus insipida* Willd. | - | 4 (0.08) | 4 |
|  | *Ficus maxima* Mill. | - | 4 (0.15) | 4 |
|  | *Ficus popenoei* Standl. | - | 1 (0.04) | 1 |
|  | *Pseudolmedia glabrata* (Liebm.) C.C. Berg | 21 (1.00) | 92 (0.81) | 113 |
|  | *Trophis mexicana* (Liebm.) Bureau | - | 2 (0.04) | 2 |
|  | *Trophis racemosa* (L.) Urb. | 3 (0.50) | 17 (0.42) | 20 |
| Myristicaceae | *Virola koschnyi* Warb. | 1 (0.25) | 17 (0.35) | 18 |
| Myrtaceae | *Eugenia acapulcensis* Steud. | 1 (0.25) | 4 (0.15) | 5 |
|  | *Eugenia aeruginea* DC. | - | 10 (0.19) | 10 |
|  | *Eugenia capuli* (Schltdl. & Cham.) Hook. & Arn. | - | 2 (0.08) | 2 |
|  | *Eugenia nigrita* Lundell | 1 (0.25) | - | 1 |
|  | *Psidium friedrichsthalianum* (O. Berg) Nied. | 3 (0.50) | 26 (0.38) | 29 |
| Nyctaginaceae | *Neea stenophylla* Standl. | - | 1 (0.04) | 1 |
| Ochnaceae | *Ouratea crassinervia* Engl. | 2 (0.50) | 3 (0.08) | 5 |
|  | *Ouratea lucens* (Kunth) Engl. | 1 (0.25) | 11 (0.23) | 12 |
| Passifloraceae | *Erblichia odorata* Seem. | 3 (0.25) | - | 3 |
| Piperaceae | *Piper aduncum* L. | 1 (0.25) | 2 (0.08) | 3 |
|  | *Piper aequale* Vahl | 14 (0.75) | 13 (0.23) | 27 |
|  | *Piper auritum* Kunth | - | 14 (0.08) | 14 |
|  | *Piper hispidum* Sw. | - | 6 (0.12) | 6 |
|  | *Piper* sp. 1 | 4 (1.00) | 13 (0.35) | 17 |
|  | *Piper* sp.2 | - | 1 (0.04) | 1 |
| Polygonaceae | *Coccoloba belizensis* Standl. | - | 15 (0.12) | 15 |
| Primulaceae | *Ardisia conzattii* S.F. Blake | - | 37 (0.23) | 37 |
|  | *Parathesis lenticellata* Lundell | 1 (0.25) | 5 (0.19) | 6 |
| Rhizophoraceae | *Cassipourea guianensis* Aubl. | 1 (0.25) | 136 (0.77) | 137 |
| Rubiaceae | *Alibertia edulis* (Rich.) A. Rich. ex DC. | - | 51 (0.54) | 51 |
|  | *Amaioua corymbosa* Kunth | 5 (0.50) | 14 (0.19) | 19 |
|  | *Blepharidium guatemalense* Standl. | - | 57 (0.54) | 57 |
|  | *Faramea occidentalis* (L.) A. Rich. | 10 (1.00) | 60 (0.38) | 70 |
|  | *Genipa americana* L. | - | 1 (0.04) | 1 |
|  | *Guettarda tikalana* Lundell | - | 2 (0.04) | 2 |
|  | *Palicourea tetragona* (Donn. Sm.) C.M. Taylor & Lorence | 4 (0.75) | 4 (0.12) | 8 |
|  | *Posoqueria latifolia* (Rudge) Schult. | 3 (0.50) | 64 (0.77) | 67 |
|  | *Psychotria carthagenensis* Jacq. | 12 (1.00) | 78 (0.73) | 90 |
|  | *Psychotria limonensis* K. Krause | 7 (0.75) | 12 (0.27) | 19 |
|  | *Psychotria trichotoma* M. Martens & Galeotti | 1 (0.25) | 12 (0.12) | 13 |
|  | *Randia armata* (Sw.) DC. | 3 (0.50) | 15 (0.27) | 18 |
|  | *Sabicea villosa* Schult. | 1 (0.25) | - | 1 |
| Rutaceae | *Zanthoxylum acuminatum* (Sw.) Sw. | - | 8 (0.23) | 8 |
|  | *Zanthoxylum caribaeum* Lam. | 2 (0.25) | 15 (0.27) | 17 |
|  | *Zanthoxylum riedelianum* Engl. | - | 13 (0.23) | 13 |
| Salicaceae | *Casearia corymbosa* Kunth | - | 15 (0.35) | 15 |
|  | *Casearia* sp. | - | 1 (0.04) | 1 |
|  | *Casearia sylvestris* Sw. | 2 (0.25) | 23 (0.31) | 25 |
|  | *Casearia tremula* (Griseb.) Griseb. ex C. Wright | 1 (0.25) | 1 (0.04) | 2 |
|  | *Lunania mexicana* Brandegee | 5 (0.75) | 19 (0.35) | 24 |
|  | *Pleuranthodendron lindenii* (Turcz.) Sleumer | - | 9 (0.19) | 9 |
|  | *Zuelania guidonia* (Sw.) Britton & Millsp. | 1 (0.25) | 32 (0.38) | 33 |
| Sapindaceae | *Allophylus camptostachys* Radlk. | - | 19 (0.12) | 19 |
|  | *Cupania belizensis* Standl. | - | 2 (0.04) | 2 |
|  | *Cupania dentata* DC. | 12 (0.25) | 79 (0.46) | 91 |
|  | *Cupania glabra* Sw. | 4 (0.50) | 4 (0.12) | 8 |
|  | *Matayba glaberrima* Radlk. | 1 (0.25) | - | 1 |
|  | *Talisia oliviformis* (Kunth) Radlk. | 2 (0.25) | 15 (0.35) | 17 |
| Sapotaceae | *Chrysophyllum mexicanum* Brandegee ex Standl. | 2 (0.50) | 8 (0.19) | 10 |
|  | *Manilkara zapota* (L.) P. Royen | 3 (0.25) | 2 (0.04) | 5 |
|  | *Pouteria campechiana* (Kunth) Baehni | - | 4 (0.15) | 4 |
|  | *Pouteria durlandii* (Standl.) Baehni | 6 (0.75) | 94 (0.92) | 100 |
|  | *Pouteria sapota* (Jacq.) H.E. Moore & Stearn | 1 (0.25) | 3 (0.08) | 4 |
|  | *Pouteria* sp. 1 | 6 (0.50) | 43 (0.58) | 49 |
|  | *Pouteria* sp. 2 | 27 (0.75) | 7 (0.04) | 34 |
| Simaroubaceae | *Quassia amara* L. | - | 34 (0.23) | 34 |
| Siparunaceae | *Siparuna thecaphora* (Poepp. & Endl.) A. DC. | 1 (0.25) | 17 (0.38) | 18 |
| Solanaceae | *Cestrum racemosum* Ruiz & Pav. | - | 3 (0.12) | 3 |
|  | *Lycianthes heteroclita* (Sendtn.) Bitter | 1 (0.25) | 1 (0.04) | 2 |
|  | *Solanum schlechtendalianum* Walp. | - | 5 (0.15) | 5 |
| Ulmaceae | *Ampelocera hottlei* (Standl.) Standl. | 43 (1.00) | 130 (0.69) | 173 |
| Urticaceae | *Cecropia obtusifolia* Bertol. | - | 1 (0.04) | 1 |
|  | *Cecropia peltata* L. | 1 (0.25) | 31 (0.42) | 32 |
|  | *Myriocarpa longipes* Liebm. | 2 (0.25) | 3 (0.08) | 5 |
|  | *Pourouma bicolor* Mart. | - | 9 (0.12) | 9 |
|  | *Urera glabriuscula* V.W. Steinm. | 3 (0.25) | 5 (0.04) | 8 |
|  | *Urera rzedowskii* V.W. Steinm. | - | 1 (0.04 | 1 |
| Violaceae | *Orthion subsessile* (Standl.) Steyerm. & Standl. | - | 6 (0.19) | 6 |
|  | *Rinorea hummelii* Sprague | 62 (0.50) | 226 (0.38) | 288 |
| Vochysiaceae | *Vochysia guatemalensis* Donn. Sm. | 3 (0.25) | 86 (0.54) | 89 |
| Morphospecies | Species 1 | - | 1 (0.04) | 1 |
|  | Species 2 | - | 1 (0.04) | 1 |
|  | Species 3 | 2 (0.50) | - | 2 |
|  | Species 4 | 6 (0.50) | 27 (0.42) | 33 |
|  | Species 5 | 3 (0.50) | 35 (0.50) | 38 |
|  | Species 6 | 10 (0.75) | 29 (0.42) | 39 |
|  | Species 7 | 8 (0.50) | 33 (0.46) | 41 |
|  | Species 8 | 1 (0.25) | 5 (0.15) | 6 |
|  | Species 9 | - | 2 (0.04) | 2 |
|  | Species 10 | 1 (0.25) | - | 1 |
|  | Species 11 | - | 2 (0.08) | 2 |
|  | Species 12 | 1 (0.25) | 27 (0.50) | 28 |
|  | Species 13 | - | 1 (0.04) | 1 |
|  | Species 14 | - | 6 (0.04) | 6 |
|  | Species 15 | - | 3 (0.04) | 3 |
|  | Species 16 | - | 1 (0.04) | 1 |
|  | Species 17 | - | 1 (0.04) | 1 |
|  | Species 18 | 1 (0.25) | - | 1 |
|  | Species 19 | 1 (0.25) | - | 1 |
|  | Species 20 | 1 (0.25) | 2 (0.08) | 3 |
|  | Species 21 | - | 5 (0.12) | 5 |
|  | Species 22 | - | 2 (0.08) | 2 |
|  | Species 23 | - | 5 (0.08) | 5 |
|  | Species 24 | - | 2 (0.04) | 2 |

**References**

1. SEMARNAT (2010). Norma Oficial Mexicana NOM-059-SEMARNAT-2010, Protección ambiental Especies nativas de México de flora y fauna silvestres-Categorías de riesgo y especificaciones para su inclusión, exclusión o cambio-Lista de especies en riesgo. Diario Oficial de la Federación. Available from http://www.semarnat.gob.mx/leyesynormas/Pages/nom_fauna.aspx. Accessed 12 July 2012.
