# Supplementary material for: Conserving Tropical Tree Diversity and Forest Structure: The Value of Small Rainforest Patches in Moderately-Managed Landscapes
Source: PLoS One. 2014 Jun 5;9(6):e98931. doi: 10.1371/journal.pone.0098931 (PMC4047051; doi:10.1371/journal.pone.0098931)
Supplement: Appendix S1 — Novelty for the Lacandon rainforest flora, Chiapas, Mexico. (DOCX) [file pone.0098931.s002.docx]

**Appendix S1. Novelty for the Lacandon rainforest flora, Chiapas, Mexico.**

*Ouratea crassinervia* Engl. (Ochnaceae)

Trees or shrubs that are characterized by its largest leaf blades chartaceous (30–54 × 6.5–13.5 cm), dull above, oblanceolate, subauriculate at the base and the marginserrulate towards the acuminate apex. This species is well known from primary and secondary forests in Nicaragua, Costa Rica, Panama and Brazil, and until now it had not been recorded for Mexico [1]. Up to now, seven species of the genus *Ouratea* had been recognized for the Mesoamerican flora in Mexico of which only three species occurred in the Lacandon region [1,2]. However, we recorded *O*. *crassinervia* in four sites of old-growth tropical rainforest in Mexico near the border with Guatemala. The voucher collection was deposited at the National Herbarium of Mexico (MEXU), Instituto de Biología, Universidad Nacional Autónoma de México, with the voucher numbers: MEXU-1360766, MEXU-1360767.

**References**

1. Whitefoord C (2011) Ochnaceae. In: Davidse G, Sousa-Sánchez M, Knapp S, Chiang F, editors. Flora Mesoamericana. Mexico City: Universidad Nacional Autónoma de México, pp. 1–26.

2. Martínez E, Ramos CH, Chiang F (1994) Lista florística de la Lacandona, Chiapas. Bol Soc Bot Mex 54:99–177.
