# Supplementary material for: Conserving Tropical Tree Diversity and Forest Structure: The Value of Small Rainforest Patches in Moderately-Managed Landscapes
Source: PLoS One. 2014 Jun 5;9(6):e98931. doi: 10.1371/journal.pone.0098931 (PMC4047051; doi:10.1371/journal.pone.0098931)
Supplement: Appendix S2 — Proportion of pioneer (i.e., early-colonizer) and persistent (i.e., late-successional and old-growth forest) plant species within different-sized forest patches and continuous forest sites in the Lacandon rainforest, Mexico. (DOCX) [file pone.0098931.s003.docx]

**Appendix S2. Proportion of pioneer (i.e., early-colonizer) and persistent (i.e., late-successional and old-growth forest) plant species within different-sized forest patches and continuous forest sites in the Lacandon rainforest, Mexico.**

Because the dominance of pioneer (or early colonizer) species can be an indicative of forest disturbance [1-5], we followed the procedure described by Arroyo-Rodríguez et al. [1] to classify the species based on their successional status: early colonizers (or pioneer species) and persistent species (both late-successional and old-growth forest species). This ecological group classification was based on information from several floras (e.g., Flora of Veracruz and Neotropical Flora), as well as several species lists [1,5-8]. Then, using a χ^2^ test for contingency tables we tested if the proportion of both groups of species differed among categories of site size. For this analysis we excluded morphospecies and those taxa identified only to genus level because we could not classify them within any of these two ecological groups.

All sites were dominated by persistent plant species (82.3% ± 6.9% of species sampled per site, mean ± SD). The proportion of ecological groups was almost identical within all categories of site size (χ^2^ = 0.96, *P* = 0.92; Figure S1).

**Figure S1. Proportion and absolute number (in boxes) of plant species belonging to different successional groups (persistent and pioneer species) within different-sized forest patches and continuous forest sites (CF) in the Lacandon rainforest, Mexico.**

**References**

1. Arroyo-Rodríguez V, Pineda E, Escobar F, Benítez-Malvido J (2009) Value of small patches in the conservation of plant-species diversity in highly fragmented rainforest. Conserv Biol 23:729–739.

2. Silva JMC, Tabarelli M (2000) Tree species impoverishment and the future flora of the Atlantic forest of northeast Brazil. Nature 404:72–74.

3. Laurance WF, Nascimento HEM, Laurance SG, Andrade A, Ribeiro JELS, Giraldo JP et al. (2006) Rapid decay of tree-community composition in Amazonian forest fragments. Proc Natl Acad Sci USA 103:19010–19014.

4. Santos BA, Peres CA, Oliveira MA, Grillo A, Alves-Costa CP, Tabarelli M (2008) Drastic erosion in functional attributes of tree assemblages in Atlantic forest fragments of northeastern Brazil. Biol Conserv 141:249–260.

5. Tabarelli M, Peres CA, Melo FPL (2012) The ʻfew winners and many losersʼ paradigm revisited: emerging prospects for tropical forest biodiversity. Biol Conserv 155:136–140.

6. Martínez-Ramos M, Alvarez-Buylla ER, Sarukhán J (1989) Tree demography and gap dynamics in a tropical rain forest. Ecology 70:555–558.

7. Arroyo-Rodríguez V, Mandujano S (2006) The importance of tropical rain forest fragments to the conservation of plant species diversity in Los Tuxtlas, Mexico. Biodivers Conserv 15:4159–4179.

8. Ibarra-Manríquez G, Martínez-Ramos M, Dirzo R, Núñez-Farfán J (1997) La vegetación. In: González-Soriano E, Dirzo R, Vogt RC, editors. Historia Natural de Los Tuxtlas. Mexico City: Universidad Nacional Autónoma de México, pp. 61–85.
